# How malaria was ‘weaponised’ by the British Army during World War I

**DOI:** 10.5281/zenodo.8203655

**Published:** 2023-08-01

**Authors:** Anton Alexander

**Affiliations:** 1 BC Business Centrum, Elscot House, Arcadia Avenue, London N3 2JU, United Kingdom.

## Abstract

During the first World War (1914-1918), the British Army found itself confronting enemy armies in several countries in which malaria potentially hampered its ability to engage with the enemy. This article contrasts how it dealt with malaria on two of these fronts, the Macedonia front and the Palestine front. One front resulted in a failure of the Army to protect itself against the disease, with the other front resulting in successful protection of its troops, enabling those troops to create a decisive victory. The paper briefly explains the major differences between the two fronts, including the different attempts to deal with the disease, and draws lessons for contemporary malaria elimination efforts.

## Introduction

In 1924, six years after the conclusion of World War I (WWI) in 1918, a paper by Captain JSK Boyd of the Royal Army Medical Corps (RAMC) was published in the RAMC journal entitled ‘The Principles of the Prophylaxis of malaria: With the Administration and other measures for their application on active service.’ [1].

It was a summary of the various lines along which malaria could be attacked and the paper referred to anti-malaria measures in use by the army whilst on active service. But the paper was incomplete and/or misleading. It appeared to minimise an event where malaria had successfully been controlled; an event that had demonstrated the necessity for thoroughness [2] in anti-malaria measures, and that without such thoroughness, malaria control would have been unlikely.

The ‘minimised’ event was the very successful malaria control which took place in 1918 on the Palestine front, in the final year of WW1, which encompassed destruction of mosquito breeding sites under the direction of an entomologist. It is puzzling why the event was treated almost as an aside in the 1924 paper, and this is reflected in the following misleading comment from the paper (page 188):

*“In Palestine, during the stationary phases of the campaign, a good measure of control was obtained, the circumstances being comparatively favourable, but as soon as the final advance took place the control broke down and the disease became rampant.”*

The comment was incorrect and provided a false impression. Control did not break down. Control in fact ceased on 19th September 1918, the day of the advance by the British Army, only because from that day onwards malaria control was no longer required as it had served the Army’s purpose. Such control had successfully protected the troops from the disease in preparation for the advance.

The 1924 paper then surprisingly proceeded to disparage larval destruction, ignoring the fact that malaria was successfully controlled on the Palestine front only through use of larval source management (page 189):

*“As far as active service is concerned larva destruction can have but a limited application. Its role is that of a permanent rather than of a temporary measure. In a country which can justly be called malarious its efficient execution involves an expenditure of capital value which could rarely be justified except on the ground of results to be reaped for years to come; to employ it in a half-hearted fashion is to court disaster. It is apt to be slow in producing results. It involves much labour, and at best is bound to be limited, often at the most important points, by enemy action. For all these reasons it cannot be considered to rank high among war-time measures. In base areas it may be possible to inaugurate suitable schemes, but elsewhere, beyond dealing with flagrant breeding places, actually in or in immediate proximity to camps for the sake of sanitary discipline and a partial increase of comfort, it is doubtful if such measures will ever be of real practical value.”*

Malaria control on the Palestine front in 1918 was the only successful significant operation on a battlefront during WWI, and yet, even in 2023, little is known of this event within the malaria community. It is as yet unknown why the 1924 paper reflected a somewhat negative attitude towards larval destruction, and which attitude seems to have travelled down time, the present malaria management attitude tending to favour instead use of bednets and/ or indoor residual spraying for malaria-control.

This paper attempts to provide a very broad overview of the Palestine-front achievement, which seems to have been overlooked or ignored as either insignificant or of no interest to the malaria community. Taking note and recognising this achievement would now be particularly important for those who have never before seriously considered treating anti-malaria work as a priority.

The 1924 Boyd paper [1] opened with:

*“The association of epidemics of malaria with military campaigns in tropical and subtropical countries has been well known for many centuries. … Many a campaign has been doomed prior to its inception … through lack of appreciation of the terrible potency of this most protean of diseases sent to a fate as inevitable as it should have been obvious.”*

and continued with:

*“… a campaign in a malarious country would be a success or failure according to the thoroughness of steps taken to protect the combatants from malaria. … In Salonica during the first malaria season there were over 30,000 cases among the British troops, and in subsequent years [3,4] the number increased rather than diminished. In Palestine, after advance from the Auja line [on 19th September] the army was decimated by malaria: whilst in East Africa from January to November, 1917, there were 21,000 cases. These examples merely serve to illustrate the deadly effect of an epidemic of malaria on a fighting force, and to emphasise the necessity for prophylaxis.”*

Presumably, the intention of providing these numbers of malaria cases was to point out the dangers of malaria. But the intention of this current paper is not to compete with numbers, but to add to the 1924 paper something that was not said; something that was missing. The reader will have noted in the above extract the words:

*“In Palestine **after** advance from the Auja line, the army was decimated by malaria, …”*

The extract is strictly correct. But it is also incomplete. What is missing from it is the fact that ***before*** the advance from the Auja line (that is, before 19th September 1918), the Palestine front had been the only successful anti-malaria operation during WWI. Until 19th September, the British army had been protected, and so the successful control of malaria had served its purpose.

**Figure 1.**
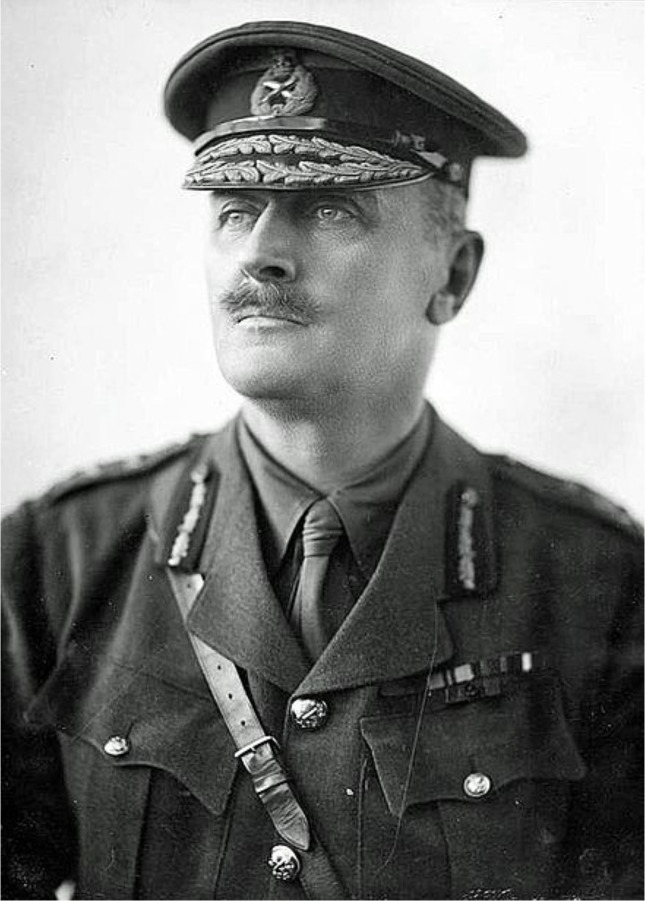
General Edmund Allenby (source: Wikipedia).

Starting the 19th of September 1918 and during the following ten days, the British Army under the command of General Allenby (Figure 1) dramatically and decisively defeated the Turkish Army on the Palestine battle front. What is little realised is that before his victory, Allenby, as a priority, had in the previous six months devoted much of the Army’s time and energy to anti-malaria measures including the destruction of most of the mosquito breeding sites within the area occupied by his Army in Palestine. He had thereby protected his troops from malaria, enabling the victorious outcome of this final and decisive battle.

The thoroughness of the steps taken to protect the British troops on the Palestine front successfully ensured the safety of those troops until the advance, and here I examine briefly some of these anti-malaria measures that were undertaken to protect the troops.

## Background to the Palestine Front

By 1917, the British Army’s mauling from the malaria experience on the Salonica (Macedonia) front had seemingly caused the British War Office to take malaria more seriously when organising a change of command that year on the Palestine front. An appreciation of the desperation and sense of helplessness of the British Army in Macedonia in dealing with the disease can be felt from a paper by Colonel C. M. Wenyon of the British Army Medical Service, published in 1921 in the RAMC journal [5], which began as follows:

*“It is now general knowledge that the military operations in Macedonia were seriously handicapped by the epidemic of malaria which attacked the troops with such devastating results during the three years of our occupation. It would seem that practically every known means of combating this terrible disease was put into operation, yet in spite of all this expenditure of energy it is doubtful if any appreciable reduction in infections took place during our stay in the country, some parts of which cannot be described as anything but pestilential. In organizing the campaign against malaria, it soon became evident that so many gaps occurred in our knowledge of the aetiology of the disease, its prevention and treatment, that [in March 1918] a special Malaria Inquiry Laboratory [6] was instituted, the duties of which were the investigation of any questions which might assist in eradicating or diminishing the incidence of the disease.”*

Palestine had been a part of the Ottoman Empire for several centuries. Palestine was already known then as notoriously malarious, rendering much of the country desolate, almost empty, either uninhabitable or sparsely populated.

In 1915, the year after the onset of WWI, the Turkish Army in Palestine attacked British positions on the Suez Canal, Egypt, but was repulsed by the British defenders. In 1916, the British Army invaded Palestine from Egypt, but attempts to capture Gaza in the south of Palestine were beaten back by the Turkish Army. In 1917, Allenby was accordingly sent to replace the commander of the British Army then in Egypt.

The British Army could have proceeded with the invasion of Palestine as if it was a conventional campaign in its attempt to defeat the Turkish Army, merely employing usual military tactics to defeat or outmanoeuvre the enemy. But its experience in Macedonia appeared to have concentrated the mind of British military headquarters and provided it with a forewarning of the dangers of malaria.

Further, events which had taken place during the previous fifty years were also to assist the British. Fortunately for the British Army, probably due to the proximity of Palestine to the Suez Canal then under construction, the Palestine Exploration Fund (PEF) had been formed. It was an organisation created in London in 1865, with Queen Victoria as its patron, ostensibly for the investigation of (and including) the archaeology, topography, geography, etc. of Palestine, the Holy Land, for Biblical purposes. The British had a colonial interest in the Suez Canal, which opened in 1869, and it has been sometimes suggested the PEF was in reality used by the British military as a cover to conceal the true military and colonial intention of the British to be familiar with all aspects, including the terrain, of Palestine. The PEF had initiated a full survey of Palestine between 1872 – 1878, the survey being conducted by British Army engineers. Thus the conditions and diseases in Palestine were already well known to the British military establishment at the onset of WWI.

By December 1917, the British Army had advanced northwards from Egypt against the Turkish army and had occupied the southern half of Palestine. However, finding itself in such a malarious area, consideration was given by the British Army to withdrawing south to a healthier position to keep the troops safe but it was decided instead to remain where it was and to deal with the disease. Accordingly, once the decision to remain had been taken, immediately from April to 19th September 1918, the British Army spent almost six months in the area it occupied thoroughly destroying the breeding sites of the anopheline mosquito, and ensuring these breeding sites remained free of larvae. Such destruction of the breeding sites was conducted under the direction of an experienced entomologist, Major E. Austen, using two thousand of its own troops and, importantly, also a great number of labourers of the Egyptian Labour Corps (ELC) for this purpose [7]. Allenby thereby effectively protected his army from malaria whilst it retrained and regrouped for the final and decisive battle against the Turkish Army.

Such protection came at a price, however. A paper [8] by one of Allenby’s medical officers later published in 1926 in the RAMC Journal noted:

*“… the Division must be prepared to lose a Brigade by holding the line. …, it was recommended that one Brigade only should be exposed to the risk [of infection], in order to save the whole Division from becoming infected. The forecast … proved a fairly accurate one. Up to September 19, the 54th and 3rd Divisions had lost just on two thousand men. If the 54th Division had remained in that area, it is probable that the figures would have been larger…”*

It might be considered that Allenby had ‘sacrificed’ these two thousand men, but it will be seen that they would anyway (if they had remained with the rest of the army) have been exposed later to infection after 19th September once the advance began.

The following [9] is an example of the thoroughness that Allenby insisted upon as part of the anti-malaria work during the six months before the final battle. Allenby arranged, in July 1918, for Colonel A. Balfour, an acknowledged expert on tropical sanitation and a future head of the London School of Hygiene and Tropical Medicine, to inspect the anti-larval work. Balfour offered some constructive suggestions and also indicated, subject to minor works he had then recommended, that he was satisfied with the anti-malaria works. Thereupon Allenby set about quietly moving his cavalry by night and concealing them in the orange groves in areas near the front line, from where the Turkish Army least expected the cavalry to attack. These orange groves would have been highly malarious before Allenby’s advance into Palestine in December 1917. Most of the mosquito breeding sites in these groves had already been destroyed by Allenby as part of his general anti-larval programme but Balfour on inspection had located a few tiny hidden breeding sites which somehow had been missed and still existed within the groves. Following Balfour’s suggestions, searches for even these tiny mosquito breeding sites were intensified, with destruction of any such breeding sites that were subsequently found.

It may be useful for the reader to appreciate the severity of malaria in Palestine at that time, including the danger the disease represented there. It will also explain the advantage that Allenby gained by the anti-malaria work when battle was to commence in September. In a published paper by one of Allenby’s senior medical officers, it was stated that had no anti-malaria work been undertaken by Allenby, his army would have ‘simply melted away’ [7]. Allenby was later to comment after his victory that he had previously been informed the incubation period for malaria was 7 – 10 days after anyone was bitten by an infected mosquito. He commented that he had taken this incubation period into account when planning his attack [10]. In fact, the British Army was to decisively defeat the Turkish Army within the 10-day incubation period time-frame from the 19th September when Allen-by’s army first crossed the front line, from the ‘healthy’ British Army area into the Turkish positions. Allenby’s calculations were proven to be correct because, approximately 10 days after the initial advance, from 1st October onwards, over 20,000 British troops, over half Allenby’s army began to collapse or ultimately die from malaria due to exposure to the bite of an infected mosquito beyond the British Army’s ‘healthy’ line. But by then, however, Allenby’s decisive victory over the Turkish Army had already been achieved.

Allenby, in effect, had ‘weaponised’ malaria because he had realised the Turkish Army beyond the front line was likely to be debilitated, suffering from the same disease to which his troops would have been exposed had no effective malaria control taken place. So when, in fact, his troops did attack and advance from their ‘healthy’ position into the Turkish malarious positions on 19th September, for a period of 10 days thereafter, the advantage of the better physical condition of the British Army troops over those of the Turkish Army was to become apparent and was mentioned in the later formal British Army report [7].

## Contribution of the Egyptian Labour Corps

In a 1930 scientific malaria publication, it was explained why it was difficult to understand at first sight how a country as sparsely settled as Palestine could have had such a disease so widely spread and epidemics so continuous. Epidemics are usually correlated with crowding. The answer was furnished by a study of the socio-economic conditions prevailing there, and that because the country was small and undeveloped, there was a constant active movement of the various population groups including the Bedouin and those on annual pilgrimages. It was noted that this movement was as effective in spreading malaria and maintaining its epidemicity as it would be in the case of any other infectious disease [11].

For Allenby on the Palestine front, anti-malaria work was to be treated as a priority [12]. He had realised he required additional labour to deal thoroughly and completely with all anti-malaria tasks, and because Palestine was so sparsely populated, almost empty, Allenby also realised he couldn’t expect native labour to be available. Rather than accepting the situation and doing nothing, he knew he had to deal with the breeding sites as a necessity, and decided therefore, as previously mentioned, to bring in great numbers of Egyptian labourers of the ELC to assist with the anti-malaria tasks.

Allenby generally wrote little of his military activities but fortunately for later historians, General Wavell, one of Allenby’s officers with him during the Palestine campaign, set about in 1936 to write of Allenby’s achievements. Wavell contacted a number of Allenby’s officers from the Palestine campaign for their memories of what and how Allenby had conducted himself during that campaign. Below is an extract of a reply to Wavell from Richard Luce, the final Director of Medical Services attached to the British Army in Palestine during the campaign with Allenby, and which reply specifically mentioned the involvement of the ELC [13]:

*“From a medical point of view, the most interesting point in his [Allenby] conduct of the Palestine campaign was his policy with regard to the Malaria Question.*

*…, he gave unstinted help towards carrying out every method of mitigating the danger [from malaria] that was put forward. Thousands of Egyptian workers were put at the disposal of the Antimalarial workers for draining marshes and training the course of streams, vast engineering projects which had never before been undertaken in the face of an enemy.*

*But the result was that the mosquito population was marvellously diminished and malaria, troublesome as it was, never became a menace to the general health or morale of the troops, who suffered infinitely less than the Turks only a short distance away across the trenches in an undoctored area,”*

Further, a quiet understated reference to the ELC was also made in the 1919 British Army Palestine Malaria Report [7]. To the reader today, the following may appear patronising and demeaning, but one hundred years ago, those were still colonial times and such language may then have been commonplace. However, the fact there was a reference at all to the ELC indicated its importance to Allenby’s army and to the huge role and contribution it made to the successful malaria control:

*“… splendid workers without whom no big undertaking was completed. Docile, patient and enduring they seem to plod on with that faith which can move mountains and at length achieve the apparently impossible.”*

Also, the following aside in the 1919 British Army Palestine Malaria report impliedly confirmed the enormous involvement of the ELC by the resulting expense [7]:

*“It is interesting to speculate what can be the future of a country such as this from a health point of view. One cannot conceive the problem which faced the Army last spring [in 1918] being undertaken by a civil authority. The expense alone would be prohibitive.”*

Dealing with the malaria on the Palestine front was a priority - the work had to be carried out regardless of expense. By contrast, Wenyon never seemed to indicate that anti-mosquito work should have been regarded as a priority on the Macedonia front.

## Use of Bednets

Wenyon in his paper [5] was to depressingly write as follows:

*“Mosquito nets, of all methods of malaria prevention in case of emergency, such as occurs when troops are moving about, are of the greatest importance. By the proper use of a net, provided the individual can remain within it all night – not always possible of course in time of war – the number of mosquito bites can be reduced to nil. It is my opinion that the mosquito net did more to prevent infection than all the other methods of malaria prevention together.”*

Wenyon did qualify his above comment with the words ‘provided the individual can remain within it all night’, but therein lay its weakness. It wasn’t possible to consider mosquito nets as an effective tool if such a tool could not be practically relied upon to be used thoroughly.

Even today, it would not be a surprise for an anti-malaria campaign to perhaps claim e.g. 70% or 80% use by inhabitants of these nets as a successful improvement. But malaria is known to be unforgiving and takes advantage of the smallest crack in anti-malaria defences. Therefore, if bednets are to remain the principal method of attack against malaria, until there can be a real, genuine 100% coverage and use of such nets by all inhabitants all the time, malaria will likely remain the scourge it is today.

A lesson from the Macedonia failure should be that for a successful outcome, all steps undertaken in malaria control must be conducted as a priority and dealt with thoroughly (including a repetitive 100% correct use of mosquito nets each and every night if necessary).

It is just not good enough to merely be seen to be doing something – anything – sometimes even ineffectively. Sadly, use of bednets today seems to generally be a ‘tired, thoughtless, default position’ within the malaria community when attempting malaria control or management.

Also, it is worth remembering Palestine’s success was not based on use of bednets but upon the timely thorough destruction of mosquito breeding sites. Such destruction was visual, it could be inspected and checked, and the result would therefore be reliable and managed accordingly. Results of the proper use of bednets, however, can sometimes be unreliable because they will always be based on trust and be completely dependent upon an inhabitant’s truthful and reliable account of how the net was used the previous night. How can such results and data be effectively managed if their reliability and accuracy can always be questioned?

A final observation - Wenyon’s above comment begins:

*“Mosquito nets, of all methods of malaria prevention in case of emergency, **such as occurs when troops are moving about**, are of the greatest importance.”*

It must be remembered that troops were also moving about on the Palestine front, yet the success there was due to destruction of the breeding sites, not reliance on bednets.

## Conclusions

It is admitted that it must have been simpler or more obvious in war-time to have made control of malaria a priority [12]. The objective of the British War Office was to defeat the enemy in battle, and the existence of malaria would have either hampered or stood in the way of that objective. Therefore, malaria had to be eventually managed, and couldn’t have been ignored.

But here lays the difficulty today. How is malaria control to be justified as a priority in peacetime? How are the malaria worker and the inhabitant to be convinced that all anti-malaria measures have to be dealt with thoroughly and without cutting corners?

The reader has to come round to treating a death from malaria as a tragedy rather than accepting such a loss as merely a fact of life, an attitude reminiscent of former colonial times. Freedom from the curse of malaria should be an obvious objective, and which is a natural aim in the affluent west. However, the affluent west does not always seem to share with other areas of the world that same malaria-free commitment it demands for itself. There is the need everywhere to somehow make good health the goal, the objective. The malaria community must not flounder as if on a ‘Macedonia front’, but should prioritise and deal with malaria control thoroughly as if on a ‘Palestine front’. As set out in a previous paper I published in 2022, there have been well-meaning attempts which appear to be trying to create a habit of a correct nightly use of bednets through awareness of the disease, but the subsequent Project Performance Evaluation of these attempts explained why such 'awareness' alone was insufficient [14].

For reasons stated above, present attempts at malaria control (or even elimination) based on bednets feels reminiscent of the old Macedonia front. There is a tendency just to accept and not to blame or question when things are being done the ‘same old way’, even when the results are disappointing or merely the same as were obtained before. It is likely only few readers of this paper will have previously known of the Palestine front. The intention of this paper is to explain how and why making the 1918 anti-malaria measures a priority caused the outcome to be so successful. Even fewer readers will be aware that it was in Palestine in 1922, the first start anywhere in the world of a successful national malaria elimination campaign began – that was made possible only because malaria elimination was treated as a priority, and innovation flowed from that. Malaria will remain with us unless and until an approach is applied to the problem that is thorough, continuous and systematic. If ridding the world of malaria truly becomes a priority, a world free of malaria need not be just a dream.
